# Medication Use Before and After Different Bariatric Surgery Procedures: Results from a Population-Based Cohort Study

**DOI:** 10.1007/s11695-025-07911-8

**Published:** 2025-05-14

**Authors:** Federico Rea, Emanuele Muraca, Gabriella Morabito, Alice Oltolini, Alessia Bongo, Gianluca Perseghin, Giovanni Corrao, Stefano Ciardullo

**Affiliations:** 1 University of Milano-Bicocca, Milan, Italy; 2 Policlinico di Monza, Monza, Italy; 3 Policlinico di Monza, Monza, Italy

**Keywords:** Surgery, Bariatric, Discontinuation, Drug

## Abstract

**Background:**

Metabolic and bariatric surgery improves most obesity-related comorbidities. Here, we evaluate the effect of different metabolic and bariatric surgery interventions on the use of medications to treat chronic conditions.

**Materials and Methods:**

This was an observational population-based cohort study performed in Lombardy, Italy. Healthcare utilization databases were used to identify all residents who underwent a metabolic and bariatric surgery procedure between 2010 and 2020 with available follow-up data for at least three years after surgery. We included patients undergoing laparoscopic sleeve gastrectomy (LSG), gastric bypass (GB), laparoscopic adjustable gastric banding (LAGB), and biliopancreatic diversion (BPD).

**Results:**

During the period 2010 to 2020, 19,450 patients (22.5% males, 13.5% with diabetes) underwent a metabolic and bariatric surgery procedure. LSG was the most commonly performed procedure (65%), followed by LAGB (19%), GB (15%), and BPD (1%). There was a significant reduction in the use of glucose-lowering and antihypertensive drugs after the procedure in all groups. Compared to LSG, the reduction in the use of glucose-lowering drugs was greater following GB (reduction at 3 years: 59 vs 65%, *p*-interaction < 0.001) and lower following LAGB (59 vs 25%, *p*-interaction < 0.001). There was a significant reduction in lipid-lowering drug use following LSG and GB (3-year reduction: 21 and 50%, *p*-interaction < 0.001), and in psychiatric drug use following LSG, GB, and LAGB (with no difference between groups). In all groups, proton pump inhibitor use increased during the first 6 months, followed by a decrease from 1 year afterward.

**Conclusion:**

The present study including a large number of patients undergoing metabolic and bariatric surgery procedures shows robust reductions in the use of glucose, blood pressure and lipid-lowering drugs at 3 years follow-up, suggesting benefits of surgery on both quality of life and healthcare costs.

**Supplementary Information:**

The online version contains supplementary material available at 10.1007/s11695-025-07911-8.

## Introduction

Metabolic and bariatric surgery represents the most effective measure to achieve and maintain significant weight loss in the long term [1, 2]. Importantly, reductions in body weight are paralleled with improvements in cardiometabolic parameters, weight-related comorbidities, quality of life, and overall survival [3, 4]. Given the high efficacy of metabolic and bariatric surgery and the rising prevalence of obesity and its associated comorbidities and costs [5], the number of surgical procedures performed has been steadily increasing in the last decades [6]. Indeed, the International Federation for the Surgery of Obesity and Metabolic Diseases estimated that there were about 390,000 bariatric procedures performed worldwide in 2018 [7].

While the mechanisms leading to weight loss are likely to be multi-factorial [8, 9], historically, bariatric procedures have been classified into restrictive (i.e., those that lead to weight loss only due to a reduction in energy intake) and malabsorptive (those that combine reduced energy intake with loss of energy within the gastrointestinal tract as a result of malabsorption) [10]. Among the former, the most commonly performed procedure is laparoscopic sleeve gastrectomy (LSG), followed by laparoscopic adjustable gastric banding (LAGB), while the most common malabsorptive procedures are represented by Roux-en-Y gastric bypass (RYGB) and one-anastomosis gastric bypass (OAGB), with biliopancreatic diversion (BPD) contributing to a limited extent in the last decade [11]. Even though they were traditionally viewed as therapeutic options for obesity per se, their role in the treatment of associated comorbidities led some authors to coin the term “metabolic surgery” [12]. Indeed, large improvements have been reported in the control of diabetes [13, 14], hypertension, and dyslipidemia [15] along with a reduction in the mean number of drugs needed to treat these conditions [16]. Generally, slightly higher response rates have been reported with the use of malabsorptive procedures [17, 18]. While greater weight loss might account for a large part of these differences in metabolic outcome and drug use, it is possible that hormonal changes that differ across different surgical procedures might play a role as well [19].

Most studies focusing on drug discontinuation rates were performed either in Asian countries or in the United States and many were either based on several high-volume metabolic and bariatric surgery hospitals or on health assurance databases [20]. To extend available evidence on the topic, we performed the present study based in Lombardy, a large region in the north of Italy. The aim was to evaluate temporal trends in the prescription of the most commonly used drugs (i.e., blood pressure, glucose, and lipid-lowering drugs as well as anti-depressants and proton pump inhibitors (PPIs)) before and after different types of metabolic and bariatric surgery interventions in a large and unselected population by means of healthcare utilization databases.

## Methods

### Setting

This study included data from healthcare utilization databases of Lombardy, a region of Italy that accounts for approximately 16% of the Italian population (almost 10 million individuals). In Italy, the whole population is covered by the National Health Service (NHS), and an automated system of databases exists in each region aimed at collecting a variety of information, including (i) an archive of residents, reporting demographic and administrative data (e.g., the date of birth, death, start and stop of NHS assistance), (ii) a database on hospital discharge records including information about primary diagnosis, co-existing conditions and performed procedures (coded according to the International Classification of Diseases-9 th Revision-Clinical Modification [ICD-9 CM] classification system), (iii) a drug dispensing database providing information on drugs delivered to the patients by community and hospital pharmacies (coded according to the Anatomical Therapeutic Chemical [ATC] classification system), and (iv) a database on co-payment exception for diagnosed chronic diseases.

Because a unique identification code was used for all databases, their linkage provided information on the complete care pathway supplied to residents for years. To preserve privacy, each individual identification code was automatically de-identified, the inverse process being allowed only to the Regional Health Authority on request from judicial authorities. Further details on healthcare utilization databases of the Lombardy region in pharmacoepidemiological studies are available in previous studies [21, 22].

### Cohort Selection

The target population included Lombardy residents aged 18 years or older. Among them, those who underwent the metabolic and bariatric surgery procedure during the period 2010 to 2020 were identified, and the dates of hospital admission and discharge were respectively defined as the “index admission” and “index discharge.” The former indicated the day of hospital admission for the procedure, while the latter indicated the date on which the patient was discharged after completing the procedure. Procedures included LSG, LAGB, BPD, and GB. ICD9-CM codes applied to identify each procedure are reported in Supplementary Table 1. Coding did not allow us to distinguish between OAGB and RYGB. We therefore considered GB as a whole.

Patients were excluded if they (i) were not beneficiaries of the NHS for at least 3 years before the index admission, and (ii) underwent the metabolic and bariatric surgery procedure within 3 years before the index admission. The remaining patients were followed from the index discharge until the earliest date among death, emigration, and 3 years after the index discharge. This was done to ensure adequate follow-up time. Moreover, supplementary analyses at 5 years after discharge were performed.

### Use of Drugs

Selected drugs dispensed in the year before the index admission and in the three-year period after the index discharge were identified. Drugs included glucose-lowering agents, antihypertensives, lipid-lowering drugs, PPIs, antidepressants, antipsychotics, and mood stabilizers. Regarding the time span after the procedure, the analyses were performed at the following time points: 6 months, 1 year, 2 years, and 3 years. Drug use was defined as having at least one dispensing during each time span.

### Covariates

Baseline characteristics included sex, age, and comorbidities (i.e., diabetes, ischemic heart disease, cerebrovascular disease, heart failure, kidney disease, respiratory disease, and cancer). In addition, the clinical status of the patients was assessed by the multisource comorbidity score, a prognostic score that has been shown to predict all-cause mortality and hospitalization of the Italian population better than some widely used conventional scores [23]. Three categories of clinical status were considered: good (0 ≤ score ≤ 4), intermediate (5 ≤ score ≤ 14), and poor (score ≥ 15).

### Data Analysis

The chi-square test was used to test for differences between patients who underwent different surgery procedures.

Summary statistics of the dispensed drugs, both before the metabolic and bariatric surgery procedure and during follow-up, were expressed as counts and percentages. Mixed models with random intercepts and an unstructured covariance structure were used to test whether the use of drugs changed during follow-up. The interaction term between time and the type of procedure was included in the model to evaluate differences in the reduction of drug use between groups.

Moreover, the chi square test was used to test for differences in the use of drugs between follow-up (i.e., at 6 months, 1 year, 2 years, and 3 years) and before the bariatric surgery procedure. The Bonferroni correction was employed to adjust for multiple comparisons. In addition, to investigate the magnitude of the reduction in drug use, the effect size statistic was estimated.

Analyses were repeated after stratification for age (≤ > 40 years) and sex. Finally, the use of glucose-lowering agents was assessed among patients with diabetes.

The Statistical Analysis System Software (version 9.4; SAS Institute, Cary, NC, USA) was used for the analyses. For all hypotheses tested, a two-tailed *p*-value < 0.05 was considered significant.

## Results

### Patients

During the period 2010 to 2020, 19,450 patients underwent the metabolic and bariatric surgery procedure. LSG was the most adopted procedure (65%), followed by LAGB (19%), GB (15%), and BPD (1%). The annual number of patients who underwent each metabolic and bariatric surgery procedure is reported in Table 1.

**Table 1:** Annual number of patients who underwent each metabolic and bariatric surgery procedure

Year	LSG (*N* = 12,758)	GB (*N* = 2967)	LAGB (*N* = 3602)	BPD (*N* = 123)
2010	389	3	324	16
2011	532	51	405	14
2012	566	158	431	17
2013	701	251	475	22
2014	880	277	483	20
2015	1026	284	364	13
2016	1550	377	402	10
2017	1653	413	307	0
2018	1841	420	218	2
2019	2289	494	139	8
2020	1331	239	54	1

*LSG* laparoscopic sleeve gastrectomy, *GB* gastric bypass, *LAGB* laparoscopic adjustable gastric banding, *BPD* biliopancreatic diversion

The characteristics of cohort members are shown in Table 2 according to the type of procedure. Overall, almost four out of five patients were women, with a higher proportion among those who underwent LAGB. Compared to patients of the other procedures, those who underwent BPD had a higher prevalence of diabetes, heart failure, and respiratory disease, and a worse clinical status.

**Table 2:** Baseline characteristics of cohort members

	LSG (*N* = 12,758)	GB (*N* = 2967)	LAGB (*N* = 3602)	BPD (*N* = 123)	*P*-value
Male sex	3010 (23.6%)	690 (23.3%)	644 (17.9%)	41 (33.3%)	< 0.001
Age class (years)					< 0.001
18–40	4763 (37.3%)	891 (30.0%)	1484 (41.2%)	43 (35.0%)	
41–60	7287 (57.1%)	1891 (63.7%)	1914 (53.1%)	71 (57.7%)	
61–65	535 (4.2%)	150 (5.1%)	148 (4.1%)	8 (6.5%)	
> 65	173 (1.4%)	35 (1.2%)	56 (1.6%)	1 (0.8%)	
Comorbidities					
Diabetes	1653 (13.0%)	591 (19.9%)	362 (10.1%)	27 (22.0%)	< 0.001
Ischemic heart disease	122 (1.0%)	28 (0.9%)	23 (0.6%)	3 (2.4%)	0.090
Cerebrovascular disease	56 (0.4%)	15 (0.5%)	16 (0.4%)	2 (1.6%)	0.267
Heart failure	42 (0.3%)	5 (0.2%)	18 (0.5%)	2 (1.6%)	0.011
Kidney disease	44 (0.3%)	7 (0.2%)	18 (0.5%)	0 (0.0%)	0.284
Respiratory disease	626 (4.9%)	158 (5.3%)	226 (6.3%)	29 (23.6%)	< 0.001
Cancer	385 (3.0%)	81 (2.7%)	118 (3.3%)	6 (4.9%)	0.379
Clinical status^a^					< 0.001
Good	9697 (76.0%)	2160 (72.8%)	2835 (78.7%)	80 (65.0%)	
Intermediate	2819 (22.1%)	762 (25.7%)	704 (19.5%)	39 (31.7%)	
Poor	242 (1.9%)	45 (1.5%)	63 (1.8%)	4 (3.3%)	

*LSG* laparoscopic sleeve gastrectomy, *GB* gastric bypass, *LAGB* laparoscopic adjustable gastric banding, *BPD* biliopancreatic diversion

^a^ The clinical status was assessed by the multisource comorbidity score (MCS). Patients were categorized as having good (0 ≤ score ≤ 4), intermediate (5 ≤ score ≤ 14) or poor (score ≥ 15) clinical status

### Use of Medications

**Fig. 1:**
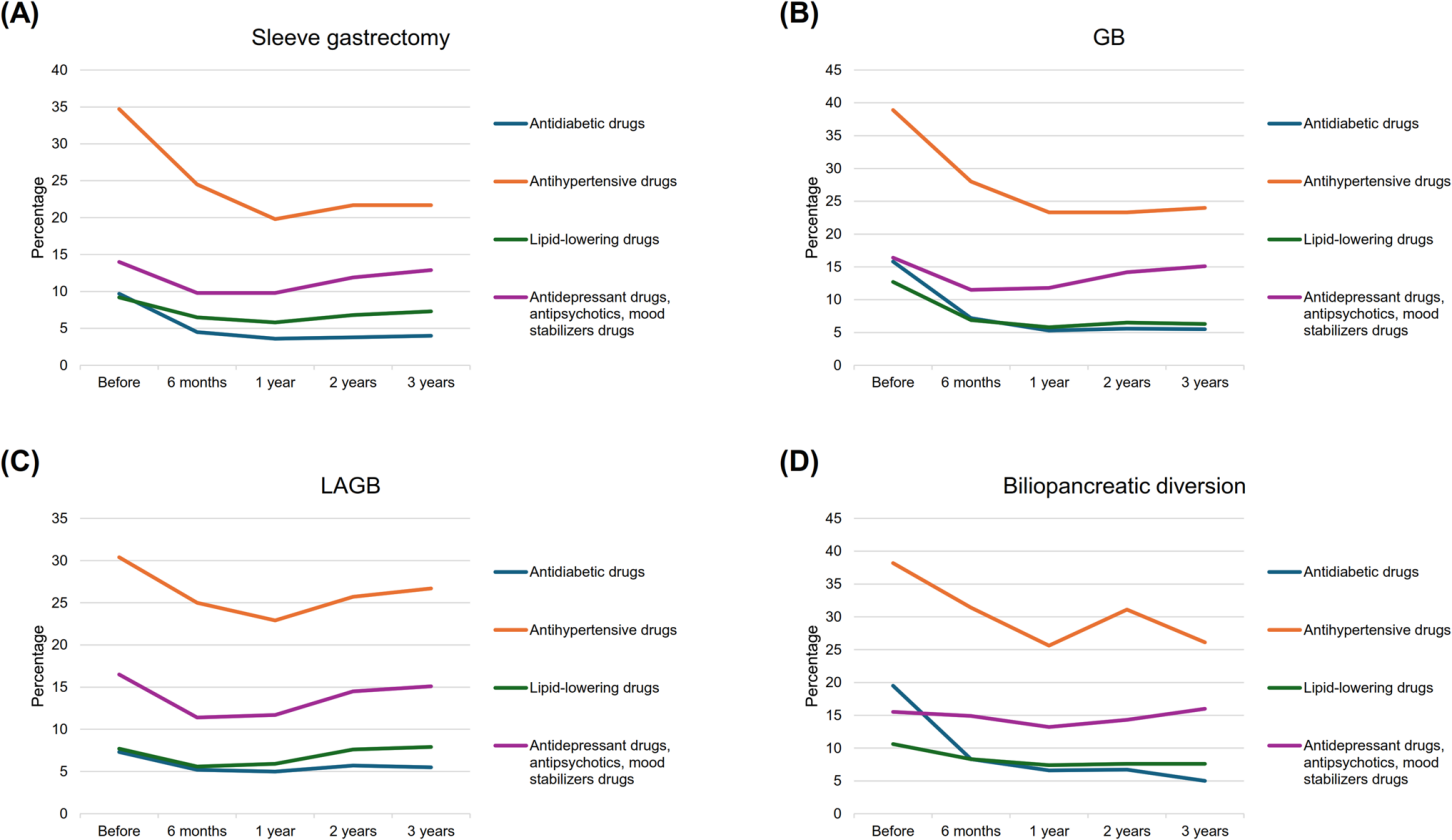
Drug treatments prescribed during the year before the metabolic and bariatric surgery procedure and during the three years after the metabolic and bariatric surgery procedure. GB, gastric bypass; LAGB, laparoscopic adjustable gastric banding

The use of selected medicaments before and after the metabolic and bariatric surgery procedure is reported in Fig. 1, Table 3, and Supplementary Tables S8, S9, and S10. There was a significant reduction in the use of glucose-lowering agents and antihypertensive drugs among all groups, although the use at 3 years was slightly greater than that at 1 year. Compared to patients who underwent LSG, there was evidence that the reduction in glucose-lowering agents use was greater among those who underwent GB (reduction at 3 years: 59 vs 65%, *p*-interaction < 0.001) and lower among those who underwent LAGB (59 vs 25%, *p*-interaction < 0.001), and the reduction in antihypertensive drugs was lower among those who underwent LAGB (37 vs 22%, *p*-interaction < 0.001). There was a significant reduction in lipid-lowering drug use among patients who underwent LSG and GB (3-year reduction: 21 and 50%, *p*-value < 0.001), and in mental drug use among those who underwent LSG, GB, and LAGB (with no difference between groups: *p*-interaction > 0.05). In all groups, there was an increase in the use of PPIs during the first six months after the procedure, followed by a decrease from 1 year afterward. Results were consistent when analyses were performed at a 5-year follow-up (Supplementary Table S7).

**Table 3:** Drug treatments prescribed during the year before the metabolic and bariatric surgery procedure and during the three years after the metabolic and bariatric surgery procedure

Metabolic and bariatric surgery procedure	Drug treatments	Before	After				*P*-trend
			6 months	1 year	2 years	3 years	
LSG	Glucose-lowering agents	1243 (9.7%)	570 (4.5%)	451 (3.6%)	484 (3.8%)	504 (4.0%)	< 0.001
	Antihypertensive drugs	4422 (34.7%)	3114 (24.5%)	2510 (19.8%)	2735 (21.7%)	2715 (21.7%)	< 0.001
	Lipid-lowering drugs	1173 (9.2%)	831 (6.5%)	735 (5.8%)	850 (6.8%)	912 (7.3%)	< 0.001
	PPIs	5446 (42.7%)	10,153 (79.8%)	4564 (36.0%)	4874 (38.7%)	4527 (36.2%)	< 0.001
	Antidepressant drugs, antipsychotics, and mood stabilizers drugs	1789 (14.0%)	1245 (9.8%)	1245 (9.8%)	1501 (11.9%)	1607 (12.9%)	< 0.001
GB	Glucose-lowering agents	468 (15.8%)	212 (7.2%)	157 (5.3%)	165 (5.6%)	159 (5.5%)	< 0.001
	Antihypertensive drugs	1153 (38.9%)	828 (28.0%)	688 (23.3%)	683 (23.3%)	698 (24.0%)	< 0.001
	Lipid-lowering drugs	376 (12.7%)	205 (6.9%)	170 (5.8%)	191 (6.5%)	184 (6.3%)	< 0.001
	PPIs	1630 (54.9%)	2330 (78.7%)	1060 (35.9%)	1215 (41.4%)	1106 (37.9%)	< 0.001
	Antidepressant drugs, antipsychotics, and mood stabilizers drugs	485 (16.4%)	339 (11.5%)	348 (11.8%)	416 (14.2%)	441 (15.1%)	0.003
LAGB	Glucose-lowering agents	262 (7.3%)	188 (5.2%)	180 (5.0%)	203 (5.7%)	195 (5.5%)	< 0.001
	Antihypertensive drugs	1096 (30.4%)	900 (25.0%)	822 (22.9%)	917 (25.7%)	945 (26.7%)	< 0.001
	Lipid-lowering drugs	276 (7.7%)	202 (5.6%)	213 (5.9%)	271 (7.6%)	279 (7.9%)	0.945
	PPIs	1507 (41.8%)	2281 (63.5%)	855 (23.8%)	1097 (30.8%)	1087 (30.7%)	< 0.001
	Antidepressant drugs, antipsychotics, and mood stabilizers drugs	594 (16.5%)	410 (11.4%)	419 (11.7%)	517 (14.5%)	535 (15.1%)	0.003
BPD	Glucose-lowering agents	24 (19.5%)	10 (8.3%)	8 (6.6%)	8 (6.7%)	6 (5.0%)	< 0.001
	Antihypertensive drugs	47 (38.2%)	38 (31.4%)	31 (25.6%)	37 (31.1%)	31 (26.1%)	0.040
	Lipid-lowering drugs	13 (10.6%)	10 (8.3%)	9 (7.4%)	9 (7.6%)	9 (7.6%)	0.349
	PPIs	67 (54.5%)	107 (88.4%)	59 (48.8%)	69 (58.0%)	68 (57.1%)	0.114
	Antidepressant drugs, antipsychotics, and mood stabilizers drugs	19 (15.5%)	18 (14.9%)	16 (13.2%)	17 (14.3%)	19 (16.0%)	0.636

*LSG* laparoscopic sleeve gastrectomy, *GB* gastric bypass, *LAGB* laparoscopic adjustable gastric banding, *BPD* biliopancreatic diversion, *PPIs* proton pump inhibitors

*P-value refers to the test for changes in drug use over the follow-up period (according to a mixed model with random intercept and an unstructured covariance structure)

### Subgroup Analyses

The results stratified for age and sex are reported in Supplementary Tables S2, S3, S4, and S5. The results described in the above paragraph did not substantially modify across strata of age and sex.

The use of glucose-lowering agents among patients with diabetes is shown in Table 4, while the baseline features of patients with diabetes are shown in Supplementary Table S6. The reduction in the overall use of antidiabetic drug treatment was also observed in all classes of antidiabetic agents. Compared to patients who underwent LSG, the decrease in the mean number of drugs was greater among those who underwent GB (reduction at 3 years: 67 vs 76%, *p*-value = < 0.001) and lower among those who underwent LAGB (67 vs 44%, *p*-value < 0.001).

**Table 4:** Antidiabetic drugs prescribed during the year before the metabolic and bariatric surgery procedure and during the three years after the metabolic and bariatric surgery procedure among patients with diabetes

Metabolic and bariatric surgery procedure	Antidiabetic drug	Before	After				p-trend
			6 months	1 year	2 years	3 years	
LSG	Metformin, *n* (%)	1113 (67.3%)	433 (26.3%)	344 (21.0%)	372 (22.9%)	376 (23.4%)	< 0.001
	DPP-4 inhibitor, *n* (%)	80 (4.8%)	27 (1.6%)	20 (1.2%)	31 (1.9%)	26 (1.6%)	< 0.001
	Sulfonylurea, *n* (%)	223 (13.5%)	50 (3.0%)	32 (2.0%)	40 (2.5%)	35 (2.2%)	< 0.001
	Pioglitazone, *n* (%)	99 (6.0%)	33 (2.0%)	18 (1.1%)	24 (1.5%)	21 (1.3%)	< 0.001
	GLP-1 RA, *n* (%)	176 (10.7%)	61 (3.7%)	49 (3.0%)	60 (3.7%)	76 (4.7%)	< 0.001
	SGLT2 inhibitor, *n* (%)	105 (6.4%)	36 (2.2%)	27 (1.7%)	34 (2.1%)	44 (2.7%)	< 0.001
	Other oral antidiabetic drugs, *n* (%)	62 (3.8%)	16 (1.0%)	13 (0.8%)	12 (0.7%)	9 (0.6%)	< 0.001
	Insulin, *n* (%)	266 (16.1%)	109 (6.6%)	92 (5.6%)	96 (5.9%)	89 (5.5%)	< 0.001
	Number of drugs, mean [SD]	1.28 [1.09]	0.46 [0.74]	0.36 [0.67]	0.41 [0.76]	0.42 [0.77]	< 0.001
GB	Metformin, *n* (%)	410 (69.4%)	155 (26.3%)	116 (19.7%)	123 (21.1%)	116 (20.0%)	< 0.001
	DPP-4 inhibitor, *n* (%)	28 (4.7%)	8 (1.4%)	8 (1.4%)	6 (1.0%)	10 (1.7%)	< 0.001
	Sulfonylurea, *n* (%)	103 (17.4%)	27 (4.6%)	9 (1.5%)	8 (1.4%)	10 (1.7%)	< 0.001
	Pioglitazone, *n* (%)	53 (9.0%)	16 (2.7%)	14 (2.4%)	9 (1.6%)	8 (1.4%)	< 0.001
	GLP-1 RA, *n* (%)	101 (17.1%)	24 (4.1%)	15 (2.6%)	16 (2.8%)	19 (3.3%)	< 0.001
	SGLT2 inhibitor, *n* (%)	49 (8.3%)	17 (2.9%)	13 (2.2%)	14 (2.4%)	11 (1.9%)	< 0.001
	Other oral antidiabetic drugs, *n* (%)	21 (3.6%)	5 (0.9%)	1 (0.2%)	2 (0.3%)	3 (0.5%)	< 0.001
	Insulin, *n* (%)	137 (23.2%)	55 (9.3%)	28 (4.8%)	39 (6.7%)	35 (6.0%)	< 0.001
	Number of drugs, mean [SD]	1.53 [1.17]	0.52 [0.81]	0.35 [0.64]	0.37 [0.67]	0.37 [0.70]	< 0.001
LAGB	Metformin, *n* (%)	221 (61.1%)	143 (39.6%)	132 (36.8%)	145 (40.6%)	134 (38.3%)	< 0.001
	DPP-4 inhibitor, *n* (%)	23 (6.4%)	14 (3.9%)	13 (3.6%)	13 (3.6%)	11 (3.1%)	0.002
	Sulfonylurea, *n* (%)	71 (19.6%)	23 (6.4%)	18 (5.0%)	17 (4.8%)	18 (5.1%)	< 0.001
	Pioglitazone, *n* (%)	30 (8.3%)	16 (4.4%)	19 (5.3%)	19 (5.3%)	16 (4.6%)	0.061
	GLP-1 RA, *n* (%)	37 (10.2%)	21 (5.8%)	18 (5.0%)	20 (5.6%)	22 (6.3%)	0.003
	SGLT2 inhibitor, *n* (%)	18 (5.0%)	9 (2.5%)	7 (2.0%)	12 (3.4%)	14 (4.0%)	0.019
	Other oral antidiabetic drugs, *n* (%)	22 (6.1%)	9 (2.5%)	7 (2.0%)	4 (1.1%)	7 (2.0%)	< 0.001
	Insulin, *n* (%)	57 (15.8%)	33 (9.1%)	34 (9.5%)	37 (10.4%)	38 (10.9%)	< 0.001
	Number of drugs, mean [SD]	1.32 [1.19]	0.74 [0.88]	0.69 [0.86]	0.75 [0.90]	0.74 [0.93]	< 0.001
BPD	Metformin, *n* (%)	22 (81.5%)	5 (18.5%)	6 (22.2%)	5 (18.5%)	4 (14.8%)	< 0.001
	DPP-4 inhibitor, *n* (%)	2 (7.4%)	0 (0.0%)	0 (0.0%)	1 (3.7%)	1 (3.7%)	0.439
	Sulfonylurea, *n* (%)	4 (14.8%)	1 (3.7%)	0 (0.0%)	0 (0.0%)	0 (0.0%)	0.053
	Pioglitazone, *n* (%)	1 (3.7%)	1 (3.7%)	1 (3.7%)	1 (3.7%)	1 (3.7%)	1.000
	GLP-1 RA, *n* (%)	5 (18.5%)	0 (0.0%)	0 (0.0%)	1 (3.7%)	1 (3.7%)	0.051
	SGLT2 inhibitor, *n* (%)	1 (3.7%)	0 (0.0%)	0 (0.0%)	0 (0.0%)	1 (3.7%)	
	Other oral antidiabetic drugs, *n* (%)	0 (0.0%)	0 (0.0%)	0 (0.0%)	1 (3.7%)	1 (3.7%)	
	Insulin, *n* (%)	9 (33.3%)	4 (14.8%)	3 (11.1%)	3 (11.1%)	3 (11.1%)	0.013
	Number of drugs, mean [SD]	1.63 [0.97]	0.41 [0.57]	0.37 [0.63]	0.44 [0.80]	0.44 [0.93]	< 0.001

*LSG* laparoscopic sleeve gastrectomy, *GB* gastric bypass *LAGB* laparoscopic adjustable gastric banding, *BPD* biliopancreatic diversion, *DPP-4* dipeptidyl peptidase 4, *GLP-1 RA* glucagon-like peptide-1 receptor agonists, *SGLT2* sodium-glucose cotransporter-2, *SD* standard deviation

*P-value refers to the test for changes in drug use over the follow-up period (according to a mixed model with random intercept and an unstructured covariance structure)

## Discussion

In the present large population-based cohort study, we made a series of observations. First, LSG was by far the most commonly performed procedure, accounting for approximately two-thirds of all bariatric interventions and females represent ~ 75% of patients receiving this intervention. Second, metabolic and bariatric surgery was associated with a significant reduction in the use of glucose, lipid, and blood pressure lowering drugs throughout the study period (i.e., 3 years), while reductions in psychiatric drugs were less pronounced and PPIs use increased in the first six months and then dropped to baseline values, irrespectively of the specific procedure. We decided to focus on PPI and psychiatric drugs as it is known that different bariatric procedures have different effects on gastroesophageal reflux disease (LSG increases its severity, while GB might attenuate it); moreover, some studies have shown increased rates of self-harm and suicidal ideation after bariatric surgery, a topic that remains controversial.

Third, compared with LSG, the reduction in glucose-lowering agent use was greater among those who underwent GB and lower among those who underwent LAGB. Fourth, the benefits of the procedure were shared by males and females, as well as younger and older patients. Fifth, among patients with diabetes, reductions were found in the use of all classes of antidiabetic agents, including insulin.

The results of the current study are generally in line with previous investigations. For instance, in a recent large study performed in the USA, Howard et al. showed a marked reduction in glucose-, blood pressure-, and lipid-lowering drugs in patients who underwent either LSG or RYGB at 5-year follow-up [20]. Similar results were also reported in a recent study from North Korea [24]. Our results confirm these trends and extend them to a European context. Regarding the effect of each specific procedure, our results are in line with data from randomized clinical trials, showing slightly higher reductions in body weight and better glycemic control in patients undergoing GB compared with LSG. For instance, in the SLEEVEPASS trial, which randomized 240 patients to either LSG or RYGB, at 10 years, patients undergoing RYGB lost more weight (% excess weight loss was 43.5% after LSG and 50.7% after RYGB) and had slightly higher rate of diabetes remission (26% in the LSG and 33% in the RYGB groups, respectively), even though the latter difference was not statistically significant [25]. Similar results were reported in a systematic review and meta-analysis of comparative studies [26]. The lower reduction in medication use following LAGB was expected, based on previous studies as well as on the lower effectiveness of this procedure on weight loss [27].

An interesting aspect was related to the use of PPIs before and after the different procedures. Data from the literature are concordant in reporting a higher risk of worsening gastroesophageal reflux disease (GERD) after LSG compared with GB [18]. In the current study, this class was the most commonly used before interventions, with approximately half of all patients taking them. We then found an increase in the use of these drugs at six months after surgery in all groups and a subsequent reduction to levels similar to baseline. It is well known that GERD is a common complication of some of these procedures, in particular SG [28].

While the reasons for this trend are unclear, we speculate that it is related to hospital protocols prescribing these drugs to most patients at hospital discharge, irrespective of symptoms, with the possibility of discontinuing them later if GERD symptoms are absent. It is therefore likely that the trend reflects clinical practice rather than disease patterns. Nonetheless, we do not have definitive data to evaluate whether this increase in PPI use was indeed clinically necessary or just a precautionary measure.

The present study has several elements of strength. First, the investigation was based on a large unselected population, which was made possible because, in Italy, a cost-free healthcare system involves virtually all citizens. Second, the drug dispensing database provided highly accurate data because pharmacists are required to report dispensing in detail to obtain reimbursement, and incorrect reports about the dispensed drugs have legal consequences [29, 30]. Third, patients who underwent metabolic and bariatric surgery procedures in the three years preceding the considered intervention were excluded. While it is still possible that patients had undergone a bariatric procedure before this time frame, the effects of the previous procedures should have stabilized after at least 3 years, and changes in medications following the index procedure can be attributed to the procedure itself.

On the same lines, some limitations need to be acknowledged. First, our database did not record drugs prescribed outside the NHS (i.e., over-the-counter medications). While it is unlikely that a significant number of patients used glucose, lipid, or blood pressure-lowering drugs for long periods without them being covered by the NHS, this could be the case for PPIs and some psychiatric medications. The use of such drugs, therefore, may be underestimated. Another significant aspect that was not considered related to adherence to drug treatment, which is known to be low for most chronic asymptomatic conditions. We will focus on this aspect in future endeavors evaluating the proportion of days covered by drug treatment after bariatric surgery. Second, information on drug use is limited to dispensing and actual drug consumption by patients could not be assessed. Similarly, data on changes in lifestyle after surgery, which might impact medication use, were not available. It is well known that some of the effects of bariatric surgery on both weight loss and metabolic comorbidities (and therefore on their treatment) are mediated by such changes. Indeed, lifestyle changes are often a significant part of the post-surgery management plan and might contribute to reductions in medication use. While it is unlikely that these were systematically different across different surgical procedures, this aspect deserves attention in future studies.

Third, because our database does not include several clinical data (e.g., body mass index and glycated hemoglobin), we cannot evaluate the impact of metabolic and bariatric surgery on these clinical endpoints. Fourth, because this is not a randomized controlled trial, our results on the comparison between groups may be affected by confounding, especially due to those characteristics not recorded in the database, such as baseline severity of comorbidities or socio-economic status. However, because the main analysis involved a within-group comparison at different time points, all factors that remained fixed during the follow-up period did not affect the most important finding of our study, i.e. the significant reduction in the use of glucose, lipid, and blood pressure lowering drugs after the procedure in each group. Finally, there are no specific ICD9-CM codes for the identification of metabolic and bariatric surgery procedures and the diagnoses could not be validated since hospital records were not available for scrutiny. Although we followed previous literature reports [31] as well as instructions from regional healthcare authorities on correct coding, some misclassification might have occurred. In addition, the distinction between OAGB and RYGB was not possible. It is noteworthy, however, to consider that these two interventions have similar effects on body weight and related comorbidities [32].

In conclusion, the present large, population-based, cohort study suggests that metabolic and bariatric surgery leads to lower use of several classes of drugs to treat obesity-related comorbidities such as diabetes, hypertension, and dyslipidemia. It also suggests greater efficacy of GB compared with LSG on glycemic outcomes and a lower impact of LAGB. Even in an era when pharmacological therapies might achieve weight loss goals similar to those achieved with surgery [33], metabolic and bariatric surgery could still represent an intervention able to improve quality of life and favorably impact healthcare costs related to drug assumption.

## Supplementary Information

Below is the link to the electronic supplementary material.

Supplementary file 1 (DOCX 57 KB)

## Data Availability

Restrictions apply to the availability of some or all data generated or analyzed during this study to preserve patient confidentiality or because they were used under license. The corresponding author will on request detail the restrictions and any conditions under which access to some data may be provided.

## Code Availability

Code is available from the corresponding author upon reasonable request.
