# Supplementary material for: Medication Use Before and After Different Bariatric Surgery Procedures: Results from a Population-Based Cohort Study
Source: Obes Surg. 2025 May 14;35(6):2240–8. doi: 10.1007/s11695-025-07911-8 (PMC12129833; doi:10.1007/s11695-025-07911-8)
Supplement: Supplementary file 1 — (DOCX 57 KB) [file 11695_2025_7911_MOESM1_ESM.docx]

**Supplementary Table S1**. Codes used to identify patients who underwent bariatric surgery procedure

|  | **Codes** |
| --- | --- |
| **Diseases** † |  |
| Severe obesity | 278.01 |
| **Procedures** † |  |
| Laparoscopic sleeve gastrectomy | 43.89, 44.99 |
| Gastric bypass | 44.38 |
| Laparoscopic adjustable gastric banding | 44.95 |
| Biliopancreatic diversion | 43.7 AND 45.91 |

^†^ According to the ICD-9-CM (International Classification of Disease, 9th Revision) system

**Supplementary Table S2**. Drug treatments prescribed during the year before the bariatric surgery procedure and during the three years after the bariatric surgery procedure among patients aged 18-40 years

| **Bariatric Surgery Procedure** | **Drug treatments** | **Before** | **After** | | | | **p-trend** |
| --- | --- | --- | --- | --- | --- | --- | --- |
|  |  |  | **6 months** | **1 year** | **2 years** | **3 years** |  |
| LSG | Glucose-lowering agents | 178 (3.7%) | 51 (1.1%) | 31 (0.7%) | 47 (1.0%) | 48 (1.0%) | <0.001 |
|  | Antihypertensive drugs | 552 (11.6%) | 325 (6.9%) | 207 (4.4%) | 247 (5.3%) | 241 (5.2%) | <0.001 |
|  | Lipid-lowering drugs | 111 (2.3%) | 90 (1.9%) | 53 (1.1%) | 58 (1.2%) | 53 (1.1%) | <0.001 |
|  | PPIs | 1,560 (32.8%) | 3,568 (75.2%) | 1,244 (26.3%) | 1,344 (28.7%) | 1,192 (25.7%) | <0.001 |
|  | Antidepressant drugs, antipsychotics, mood stabilizers drugs | 416 (8.7%) | 273 (5.8%) | 266 (5.6%) | 341 (7.3%) | 367 (7.9%) | 0.001 |
| GB | Glucose-lowering agents | 59 (6.6%) | 17 (1.9%) | 10 (1.1%) | 11 (1.3%) | 12 (1.4%) | <0.001 |
|  | Antihypertensive drugs | 126 (14.1%) | 79 (8.9%) | 58 (6.6%) | 65 (7.4%) | 68 (7.8%) | <0.001 |
|  | Lipid-lowering drugs | 14 (1.6%) | 8 (0.9%) | 6 (0.7%) | 10 (1.1%) | 8 (0.9%) | 0.087 |
|  | PPIs | 402 (45.1%) | 664 (74.7%) | 241 (27.2%) | 286 (32.5%) | 239 (27.4%) | <0.001 |
|  | Antidepressant drugs, antipsychotics, mood stabilizers drugs | 95 (10.7%) | 61 (6.9%) | 66 (7.5%) | 85 (9.7%) | 93 (10.7%) | 0.447 |
| LAGB | Glucose-lowering agents | 36 (2.4%) | 18 (1.2%) | 19 (1.3%) | 29 (2.0%) | 23 (1.6%) | 0.111 |
|  | Antihypertensive drugs | 143 (9.6%) | 108 (7.3%) | 81 (5.5%) | 93 (6.3%) | 104 (7.2%) | <0.001 |
|  | Lipid-lowering drugs | 23 (1.6%) | 14 (0.9%) | 18 (1.2%) | 17 (1.2%) | 13 (0.9%) | 0.297 |
|  | PPIs | 490 (33.0%) | 823 (55.5%) | 219 (14.8%) | 310 (21.1%) | 284 (19.5%) | <0.001 |
|  | Antidepressant drugs, antipsychotics, mood stabilizers drugs | 144 (9.7%) | 102 (6.9%) | 102 (6.9%) | 124 (8.5%) | 137 (9.4%) | 0.138 |
| BPD | Glucose-lowering agents | 4 (9.3%) | 1 (2.4%) | 0 (0.0%) | 1 (2.4%) | 0 (0.0%) | 0.082 |
|  | Antihypertensive drugs | 8 (18.6%) | 5 (11.9%) | 4 (9.5%) | 5 (11.9%) | 5 (11.9%) | 0.188 |
|  | Lipid-lowering drugs | 3 (7.0%) | 2 (4.8%) | 2 (4.8%) | 3 (7.1%) | 3 (7.1%) | 0.989 |
|  | PPIs | 20 (46.5%) | 36 (85.7%) | 18 (42.9%) | 22 (52.4%) | 20 (47.6%) | 0.466 |
|  | Antidepressant drugs, antipsychotics, mood stabilizers drugs | 2 (4.7%) | 2 (4.8%) | 2 (4.8%) | 3 (7.1%) | 6 (14.3%) | 0.596 |

LSG: Laparoscopic Sleeve Gastrectomy; GB: Gastric Bypass; LAGB: Laparoscopic Adjustable Gastric Banding; BPD: Biliopancreatic Diversion; PPIs: Proton Pump Inhibitors

**Supplementary Table S3**. Drug treatments prescribed during the year before the bariatric surgery procedure and during the three years after the bariatric surgery procedure among patients aged >40 years

| **Bariatric Surgery Procedure** | **Drug treatments** | **Before** | **After** | | | | **p-trend** |
| --- | --- | --- | --- | --- | --- | --- | --- |
|  |  |  | **6 months** | **1 year** | **2 years** | **3 years** |  |
| LSG | Glucose-lowering agents | 1,065 (13.3%) | 519 (6.5%) | 420 (5.3%) | 437 (5.5%) | 456 (5.8%) | <0.001 |
|  | Antihypertensive drugs | 3,870 (48.4%) | 2,789 (35.0%) | 2,303 (28.9%) | 2,488 (31.5%) | 2,474 (31.5%) | <0.001 |
|  | Lipid-lowering drugs | 1,062 (13.3%) | 741 (9.3%) | 682 (8.6%) | 792 (10.0%) | 859 (10.9%) | <0.001 |
|  | PPIs | 3,886 (48.6%) | 6,585 (82.5%) | 3,320 (41.7%) | 3,530 (44.6%) | 3,335 (42.4%) | <0.001 |
|  | Antidepressant drugs, antipsychotics, mood stabilizers drugs | 1,373 (17.2%) | 972 (12.2%) | 979 (12.3%) | 1,160 (14.7%) | 1,240 (15.8%) | <0.001 |
| GB | Glucose-lowering agents | 409 (19.7%) | 195 (9.4%) | 147 (7.1%) | 154 (7.5%) | 147 (7.2%) | <0.001 |
|  | Antihypertensive drugs | 1,027 (49.5%) | 749 (36.2%) | 630 (30.5%) | 618 (30.1%) | 630 (30.9%) | <0.001 |
|  | Lipid-lowering drugs | 362 (17.4%) | 197 (9.5%) | 164 (7.9%) | 181 (8.8%) | 176 (8.6%) | <0.001 |
|  | PPIs | 1,228 (59.2%) | 1,666 (80.4%) | 819 (39.6%) | 929 (45.3%) | 867 (42.5%) | <0.001 |
|  | Antidepressant drugs, antipsychotics, mood stabilizers drugs | 390 (18.8%) | 278 (13.4%) | 282 (13.7%) | 331 (16.1%) | 348 (17.0%) | <0.001 |
| LAGB | Glucose-lowering agents | 226 (10.7%) | 170 (8.1%) | 161 (7.6%) | 174 (8.3%) | 172 (8.2%) | <0.001 |
|  | Antihypertensive drugs | 953 (45.0%) | 792 (37.5%) | 741 (35.1%) | 824 (39.2%) | 841 (40.3%) | <0.001 |
|  | Lipid-lowering drugs | 253 (12.0%) | 188 (8.9%) | 195 (9.3%) | 254 (12.1%) | 266 (12.8%) | 0.576 |
|  | PPIs | 1,017 (48.0%) | 1,458 (69.0%) | 636 (30.2%) | 787 (37.5%) | 803 (38.5%) | <0.001 |
|  | Antidepressant drugs, antipsychotics, mood stabilizers drugs | 450 (21.3%) | 308 (14.6%) | 317 (15.0%) | 393 (18.7%) | 398 (19.1%) | 0.007 |
| BPD | Glucose-lowering agents | 20 (25.0%) | 9 (11.4%) | 8 (10.1%) | 7 (9.1%) | 6 (7.8%) | 0.001 |
|  | Antihypertensive drugs | 39 (48.8%) | 33 (41.8%) | 27 (34.2%) | 32 (41.6%) | 26 (33.8%) | 0.095 |
|  | Lipid-lowering drugs | 10 (12.5%) | 8 (10.1%) | 7 (8.9%) | 6 (7.8%) | 6 (7.8%) | 0.286 |
|  | PPIs | 47 (58.8%) | 71 (89.9%) | 41 (51.9%) | 47 (61.0%) | 48 (62.3%) | 0.145 |
|  | Antidepressant drugs, antipsychotics, mood stabilizers drugs | 17 (21.3%) | 16 (20.3%) | 14 (17.7%) | 14 (18.2%) | 13 (16.9%) | 0.410 |

LSG: Laparoscopic Sleeve Gastrectomy; GB: Gastric Bypass; LAGB: Laparoscopic Adjustable Gastric Banding; BPD: Biliopancreatic Diversion; PPIs: Proton Pump Inhibitors

**Supplementary Table S4**. Drug treatments prescribed during the year before the bariatric surgery procedure and during the three years after the bariatric surgery procedure among men

| **Bariatric Surgery Procedure** | **Drug treatments** | **Before** | **After** | | | | **p-trend** |
| --- | --- | --- | --- | --- | --- | --- | --- |
|  |  |  | **6 months** | **1 year** | **2 years** | **3 years** |  |
| LSG | Glucose-lowering agents | 471 (15.7%) | 218 (7.3%) | 169 (5.7%) | 182 (6.1%) | 170 (5.8%) | <0.001 |
|  | Antihypertensive drugs | 1,352 (44.9%) | 1,009 (33.7%) | 786 (26.3%) | 818 (27.6%) | 798 (27.2%) | <0.001 |
|  | Lipid-lowering drugs | 463 (15.4%) | 318 (10.6%) | 259 (8.7%) | 283 (9.6%) | 290 (9.9%) | <0.001 |
|  | PPIs | 1,248 (41.5%) | 2,350 (78.4%) | 969 (32.5%) | 1,026 (34.6%) | 919 (31.4%) | <0.001 |
|  | Antidepressant drugs, antipsychotics, mood stabilizers drugs | 256 (8.5%) | 182 (6.1%) | 188 (6.3%) | 221 (7.5%) | 243 (8.3%) | 0.088 |
| GB | Glucose-lowering agents | 183 (26.5%) | 78 (11.4%) | 56 (8.2%) | 64 (9.4%) | 60 (8.9%) | <0.001 |
|  | Antihypertensive drugs | 367 (53.2%) | 274 (39.9%) | 213 (31.1%) | 201 (29.6%) | 198 (29.4%) | <0.001 |
|  | Lipid-lowering drugs | 152 (22.0%) | 83 (12.1%) | 70 (10.2%) | 77 (11.3%) | 72 (10.7%) | <0.001 |
|  | PPIs | 354 (51.3%) | 523 (76.1%) | 225 (32.8%) | 256 (37.7%) | 228 (33.8%) | <0.001 |
|  | Antidepressant drugs, antipsychotics, mood stabilizers drugs | 63 (9.1%) | 57 (8.3%) | 58 (8.5%) | 73 (10.7%) | 71 (10.5%) | 0.034 |
| LAGB | Glucose-lowering agents | 83 (12.9%) | 66 (10.3%) | 59 (9.2%) | 65 (10.2%) | 62 (9.8%) | <0.001 |
|  | Antihypertensive drugs | 269 (41.8%) | 230 (35.9%) | 188 (29.4%) | 206 (32.3%) | 219 (34.5%) | <0.001 |
|  | Lipid-lowering drugs | 98 (15.2%) | 69 (10.8%) | 70 (10.9%) | 83 (13.0%) | 83 (13.1%) | 0.007 |
|  | PPIs | 284 (44.1%) | 399 (62.3%) | 155 (24.2%) | 174 (27.3%) | 164 (25.9%) | <0.001 |
|  | Antidepressant drugs, antipsychotics, mood stabilizers drugs | 65 (10.1%) | 48 (7.5%) | 45 (7.0%) | 62 (9.7%) | 66 (10.4%) | 0.651 |
| BPD | Glucose-lowering agents | 10 (24.4%) | 5 (12.5%) | 3 (7.5%) | 3 (7.7%) | 3 (7.7%) | 0.006 |
|  | Antihypertensive drugs | 21 (51.2%) | 15 (37.5%) | 11 (27.5%) | 12 (30.8%) | 10 (25.6%) | 0.008 |
|  | Lipid-lowering drugs | 7 (17.1%) | 4 (10.0%) | 5 (12.5%) | 4 (10.3%) | 4 (10.3%) | 0.447 |
|  | PPIs | 26 (63.4%) | 33 (82.5%) | 21 (52.5%) | 24 (61.5%) | 22 (56.4%) | 0.190 |
|  | Antidepressant drugs, antipsychotics, mood stabilizers drugs | 8 (19.5%) | 8 (20.0%) | 7 (17.5%) | 5 (12.8%) | 7 (18.0%) | 0.259 |

LSG: Laparoscopic Sleeve Gastrectomy; GB: Gastric Bypass; LAGB: Laparoscopic Adjustable Gastric Banding; BPD: Biliopancreatic Diversion; PPIs: Proton Pump Inhibitors

**Supplementary Table S5**. Drug treatments prescribed during the year before the bariatric surgery procedure and during the three years after the bariatric surgery procedure among women

| **Bariatric Surgery Procedure** | **Drug treatments** | **Before** | **After** | | | | **p-trend** |
| --- | --- | --- | --- | --- | --- | --- | --- |
|  |  |  | **6 months** | **1 year** | **2 years** | **3 years** |  |
| LSG | Glucose-lowering agents | 772 (7.9%) | 352 (3.6%) | 282 (2.9%) | 302 (3.1%) | 334 (3.5%) | <0.001 |
|  | Antihypertensive drugs | 3,070 (31.5%) | 2,105 (21.6%) | 1,724 (17.8%) | 1,917 (19.9%) | 1,917 (20.0%) | <0.001 |
|  | Lipid-lowering drugs | 710 (7.3%) | 513 (5.3%) | 476 (4.9%) | 567 (5.9%) | 622 (6.5%) | <0.001 |
|  | PPIs | 4,198 (43.1%) | 7,803 (80.2%) | 3,595 (37.1%) | 3,848 (40.0%) | 3,608 (37.7%) | <0.001 |
|  | Antidepressant drugs, antipsychotics, mood stabilizers drugs | 1,533 (15.7%) | 1,063 (10.9%) | 1,057 (10.9%) | 1,280 (13.3%) | 1,364 (14.3%) | <0.001 |
| GB | Glucose-lowering agents | 285 (12.5%) | 134 (5.9%) | 101 (4.5%) | 101 (4.5%) | 99 (4.4%) | <0.001 |
|  | Antihypertensive drugs | 786 (34.5%) | 554 (24.4%) | 475 (21.0%) | 482 (21.4%) | 500 (22.3%) | <0.001 |
|  | Lipid-lowering drugs | 224 (9.8%) | 122 (5.4%) | 100 (4.4%) | 114 (5.1%) | 112 (5.0%) | <0.001 |
|  | PPIs | 1,276 (56.0%) | 1,807 (79.5%) | 835 (36.9%) | 959 (42.6%) | 878 (39.2%) | <0.001 |
|  | Antidepressant drugs, antipsychotics, mood stabilizers drugs | 422 (18.5%) | 282 (12.4%) | 290 (12.8%) | 343 (15.2%) | 370 (16.5%) | <0.001 |
| LAGB | Glucose-lowering agents | 179 (6.1%) | 122 (4.1%) | 121 (4.1%) | 138 (4.7%) | 133 (4.6%) | <0.001 |
|  | Antihypertensive drugs | 827 (28.0%) | 670 (22.7%) | 634 (21.5%) | 711 (24.3%) | 726 (25.0%) | <0.001 |
|  | Lipid-lowering drugs | 178 (6.0%) | 133 (4.5%) | 143 (4.9%) | 188 (6.4%) | 196 (6.7%) | 0.057 |
|  | PPIs | 1,223 (41.4%) | 1,882 (63.7%) | 700 (23.8%) | 923 (31.5%) | 923 (31.8%) | <0.001 |
|  | Antidepressant drugs, antipsychotics, mood stabilizers drugs | 529 (17.9%) | 362 (12.3%) | 374 (12.7%) | 455 (15.5%) | 469 (16.1%) | 0.002 |
| BPD | Glucose-lowering agents | 14 (17.1%) | 5 (6.2%) | 5 (6.2%) | 5 (6.3%) | 3 (3.8%) | 0.007 |
|  | Antihypertensive drugs | 26 (31.7%) | 23 (28.4%) | 20 (24.7%) | 25 (31.3%) | 21 (26.3%) | 0.664 |
|  | Lipid-lowering drugs | 6 (7.3%) | 6 (7.4%) | 4 (4.9%) | 5 (6.3%) | 5 (6.3%) | 0.569 |
|  | PPIs | 41 (50.0%) | 74 (91.4%) | 38 (46.9%) | 45 (56.3%) | 46 (57.5%) | 0.277 |
|  | Antidepressant drugs, antipsychotics, mood stabilizers drugs | 11 (13.4%) | 10 (12.4%) | 9 (11.1%) | 12 (15.0%) | 12 (15.0%) | 0.825 |

LSG: Laparoscopic Sleeve Gastrectomy; GB: Roux-en-Y Gastric Bypass; LAGB: Laparoscopic Adjustable Gastric Banding; BPD: Biliopancreatic Diversion; PPIs: Proton Pump Inhibitors

**Supplementary Table S6**. Baseline characteristics of cohort members with diabetes

|  | **LSG**  **(N=1,653)** | **GB**  **(N=591)** | **LAGB**  **(N=362)** | **BPD**  **(N=27)** | **p-value** |
| --- | --- | --- | --- | --- | --- |
| **Male sex** | 576 (34.9%) | 213 (36.0%) | 101 (27.9%) | 11 (40.7%) | 0.044 |
| **Age class (years)** |  |  |  |  | 0.861 |
| 18 – 40 | 289 (17.5%) | 98 (16.6%) | 65 (18.0%) | 5 (18.5%) |  |
| 41 – 60 | 1,120 (67.8%) | 418 (70.7%) | 242 (66.8%) | 19 (70.4%) |  |
| 61 – 65 | 181 (10.9%) | 58 (9.8%) | 38 (10.5%) | 3 (11.1%) |  |
| > 65 | 63 (3.8%) | 17 (2.9%) | 17 (4.7%) | 0 (0.0%) |  |
| **Comorbidities** |  |  |  |  |  |
| Ischemic heart disease | 45 (2.7%) | 18 (3.1%) | 6 (1.7%) | 2 (7.4%) | 0.256 |
| Cerebrovascular disease | 19 (1.2%) | 6 (1.0%) | 2 (0.6%) | 2 (7.4%) | 0.012 |
| Heart failure | 21 (1.3%) | 3 (0.5%) | 8 (2.2%) | 0 (0.0%) | 0.121 |
| Kidney disease | 16 (1.0%) | 5 (0.9%) | 8 (2.2%) | 0 (0.0%) | 0.171 |
| Respiratory disease | 137 (8.3%) | 38 (6.4%) | 45 (12.4%) | 10 (37.0%) | <0.001 |
| Cancer | 80 (4.8%) | 18 (3.1%) | 18 (5.0%) | 2 (7.4%) | 0.252 |
| **Clinical status**^a^ |  |  |  |  | 0.115 |
| Good | 662 (40.0%) | 230 (38.9%) | 149 (41.2%) | 6 (22.2%) |  |
| Intermediate | 876 (53.0%) | 335 (56.7%) | 193 (53.3%) | 18 (66.7%) |  |
| Poor | 115 (7.0%) | 26 (4.4%) | 20 (5.5%) | 3 (11.1%) |  |

LSG: Laparoscopic Sleeve Gastrectomy; GB: Gastric Bypass; LAGB: Laparoscopic Adjustable Gastric Banding; BPD: Biliopancreatic Diversion

^a^ The clinical status was assessed by the Multisource Comorbidity Score (MCS). Patients were categorized as having good (0 ≤ score ≤ 4), intermediate (5 ≤ score ≤ 14) or poor (score ≥ 15) clinical status.

**Supplementary Table S7** Drug treatments prescribed during the year before the bariatric surgery procedure and during the three years after the bariatric surgery procedure.

| **Bariatric Surgery Procedure** | **Drug treatments** | **Before** | **After** | | | |  |  |  |
| --- | --- | --- | --- | --- | --- | --- | --- | --- | --- |
|  |  |  | **6 months** | **1 year** | **2 years** | **3 years** | **4 years** | **5 years** | **p-trend** |
| Sleeve gastrectomy | Antidiabetic drugs | 9.7 | 4.5 | 3.6 | 3.8 | 4.0 | 4.5 | 4.6 | <0.001 |
|  | Antihypertensive drugs | 34.7 | 24.5 | 19.8 | 21.7 | 21.7 | 23.1 | 24.4 | <0.001 |
|  | Lipid-lowering drugs | 9.2 | 6.5 | 5.8 | 6.8 | 7.3 | 7.8 | 8.4 | 0.214 |
|  | Proton pump inhibitors | 42.7 | 79.8 | 36.0 | 38.7 | 36.2 | 37.0 | 37.8 | <0.001 |
|  | Antidepressant drugs, antipsychotics, mood stabilizers drugs | 14.0 | 9.8 | 9.8 | 11.9 | 12.9 | 13.4 | 14.2 | <0.001 |
| RYGB | Antidiabetic drugs | 15.8 | 7.2 | 5.3 | 5.6 | 5.5 | 6.0 | 6.4 | <0.001 |
|  | Antihypertensive drugs | 38.9 | 28.0 | 23.3 | 23.3 | 24.0 | 23.8 | 23.6 | <0.001 |
|  | Lipid-lowering drugs | 12.7 | 6.9 | 5.8 | 6.5 | 6.3 | 6.0 | 6.3 | <0.001 |
|  | Proton pump inhibitors | 54.9 | 78.7 | 35.9 | 41.4 | 37.9 | 37.7 | 35.9 | <0.001 |
|  | Antidepressant drugs, antipsychotics, mood stabilizers drugs | 16.4 | 11.5 | 11.8 | 14.2 | 15.1 | 15.7 | 16.6 | <0.001 |
| LAGB | Antidiabetic drugs | 7.3 | 5.2 | 5.0 | 5.7 | 5.5 | 5.7 | 6.1 | 0.127 |
|  | Antihypertensive drugs | 30.4 | 25.0 | 22.9 | 25.7 | 26.7 | 26.9 | 28.0 | 0.736 |
|  | Lipid-lowering drugs | 7.7 | 5.6 | 5.9 | 7.6 | 7.9 | 8.8 | 9.0 | <0.001 |
|  | Proton pump inhibitors | 41.8 | 63.5 | 23.8 | 30.8 | 30.7 | 32.4 | 34.1 | <0.001 |
|  | Antidepressant drugs, antipsychotics, mood stabilizers drugs | 16.5 | 11.4 | 11.7 | 14.5 | 15.1 | 15.8 | 15.5 | <0.001 |
| Biliopancreatic diversion | Antidiabetic drugs | 19.5 | 8.3 | 6.6 | 6.7 | 5.0 | 7.8 | 7.5 | <0.001 |
|  | Antihypertensive drugs | 38.2 | 31.4 | 25.6 | 31.1 | 26.1 | 28.5 | 30.8 | 0.031 |
|  | Lipid-lowering drugs | 10.6 | 8.3 | 7.4 | 7.6 | 7.6 | 9.5 | 9.4 | 0.95 |
|  | Proton pump inhibitors | 54.5 | 88.4 | 48.8 | 58.0 | 57.1 | 52.6 | 50.5 | 0.001 |
|  | Antidepressant drugs, antipsychotics, mood stabilizers drugs | 15.5 | 14.9 | 13.2 | 14.3 | 16.0 | 15.5 | 21.5 | 0.146 |

**Supplementary Table S8.** Estimated change, and associated confidence interval (CI), in the use of drug treatments three years after the bariatric surgery procedure vs the year before.

| **Bariatric Surgery Procedure** | **Drug treatments** | **Change (%)** | **95% CI** |
| --- | --- | --- | --- |
| LSG | Glucose-lowering agents | -58.17 | -61.01; -55.11 |
|  | Antihypertensive drugs | -37.40 | -39.08; -35.68 |
|  | Lipid-lowering drugs | -20.73 | -25.03; -16.18 |
|  | PPIs | -15.18 | -17.41; -12.90 |
|  | Antidepressant drugs, antipsychotics, mood stabilizers drugs | -8.56 | -12.13; -4.85 |
| GB | Glucose-lowering agents | -65.43 | -69.68; -60.59 |
|  | Antihypertensive drugs | -38.44 | -41.59; -35.11 |
|  | Lipid-lowering drugs | -50.03 | -55.50; -43.90 |
|  | PPIs | -30.98 | -34.32; -27.46 |
|  | Antidepressant drugs, antipsychotics, mood stabilizers drugs | -7.75 | -14.50; -0.47 |
| LAGB | Glucose-lowering agents | -23.24 | -30.75; -14.93 |
|  | Antihypertensive drugs | -12.39 | -15.93; -8.70 |
|  | Lipid-lowering drugs | 3.61 | -5.91; 14.11 |
|  | PPIs | -26.62 | -30.46; -22.57 |
|  | Antidepressant drugs, antipsychotics, mood stabilizers drugs | -8.49 | -14.53; -2.02 |
| BPD | Glucose-lowering agents | -75.02 | -87.80; -48.84 |
|  | Antihypertensive drugs | -32.45 | -47.45; -13.16 |
|  | Lipid-lowering drugs | -26.32 | -57.35; 27.29 |
|  | PPIs | 4.85 | -14.25; 28.20 |
|  | Antidepressant drugs, antipsychotics, mood stabilizers drugs | 4.60 | -28.12; 52.21 |

LSG: Laparoscopic Sleeve Gastrectomy; GB: Gastric Bypass; LAGB: Laparoscopic Adjustable Gastric Banding; BPD: Biliopancreatic Diversion; PPIs: Proton Pump Inhibitors

**Supplementary Table S9.** Differences, and confidence intervals (CI), between bariatric surgery procedures in the three-year drug use change.

| **Drug treatments** | **Comparison of surgery procedures** | **Difference in drug use change (%)** | **95% CI*** |
| --- | --- | --- | --- |
| Glucose-lowering agents | BPD vs GB | -8.32 | -19.14; 33.54 |
|  | LAGB vs GB | 39.06 | 35.00; 43.41 |
|  | BPD vs LAGB | -47.38 | -54.13; -9.87 |
|  | BPD vs LSG | -15.20 | -28.01; 29.58 |
|  | GB vs LSG | -6.87 | -8.88; -3.96 |
|  | LAGB vs LSG | 32.18 | 26.12; 39.45 |
| Antihypertensive drugs | BPD vs GB | 8.56 | -8.90; 35.81 |
|  | LAGB vs GB | 25.96 | 25.66; 26.18 |
|  | BPD vs LAGB | -17.40 | -34.56; 9.63 |
|  | BPD vs LSG | 6.64 | -13.01; 36.36 |
|  | GB vs LSG | -1.92 | -4.11; 0.55 |
|  | LAGB vs LSG | 24.04 | 21.55; 26.73 |
| Lipid-lowering drugs | BPD vs GB | 23.12 | -9.92; 108.65 |
|  | LAGB vs GB | 52.98 | 47.34; 59.16 |
|  | BPD vs LAGB | -29.85 | -57.26; 49.49 |
|  | BPD vs LSG | -6.66 | -41.31; 81.47 |
|  | GB vs LSG | -29.78 | -31.39; -27.18 |
|  | LAGB vs LSG | 23.20 | 15.95; 31.98 |
| PPIs | BPD vs GB | 35.52 | 12.86; 67.69 |
|  | LAGB vs GB | 4.55 | 3.88; 5.31 |
|  | BPD vs LAGB | 30.96 | 8.98; 62.38 |
|  | BPD vs LSG | 20.07 | -4.36; 54.29 |
|  | GB vs LSG | -15.45 | -17.22; -13.41 |
|  | LAGB vs LSG | -10.90 | -13.35; -8.10 |
| Antidepressant drugs, antipsychotics, mood stabilizers drugs | BPD vs GB | 10.62 | -23.21; 76.18 |
|  | LAGB vs GB | -0.76 | -2.13; 0.35 |
|  | BPD vs LAGB | 11.37 | -23.56; 78.32 |
|  | BPD vs LSG | 11.24 | -27.38; 82.54 |
|  | GB vs LSG | 0.63 | -4.17; 6.35 |
|  | LAGB vs LSG | -0.13 | -3.81; 4.22 |

6LSG: Laparoscopic Sleeve Gastrectomy; GB: Gastric Bypass; LAGB: Laparoscopic Adjustable Gastric Banding; BPD: Biliopancreatic Diversion; PPIs: Proton Pump Inhibitors

*Tukey correction was employed to adjust for multiple comparisons

**Supplementary Table S10.** Estimated change in the use of drug treatments six months, one year, two years and three years after the bariatric surgery procedure vs the year before.

| **Bariatric surgery procedures** | **Drug treatments** | **Change (%) in drug use vs baseline (p-value*)** | | | |
| --- | --- | --- | --- | --- | --- |
|  |  | **6 months** | **1 year** | **2 years** | **3 years** |
| LSG | Glucose-lowering agents | -53.88 (<0.001) | -63.38 (<0.001) | -60.40 (<0.001) | -58.34 (<0.001) |
|  | Antihypertensive drugs | -29.38 (<0.001) | -42.96 (<0.001) | -37.46 (<0.001) | -37.42 (<0.001) |
|  | Lipid-lowering drugs | -28.98 (<0.001) | -37.05 (<0.001) | -26.94 (<0.001) | -20.81 (<0.001) |
|  | PPIs | 86.90 (<0.001) | -15.73 (<0.001) | -9.36 (<0.001) | -15.23 (<0.001) |
|  | Antidepressant drugs, antipsychotics, mood stabilizers drugs | -30.28 (<0.001) | -30.05 (<0.001) | -14.83 (<0.001) | -8.41 (<0.001) |
| GB | Glucose-lowering agents | -54.50 (<0.001) | -66.31 (<0.001) | -64.29 (<0.001) | -65.55 (<0.001) |
|  | Antihypertensive drugs | -28.00 (<0.001) | -40.09 (<0.001) | -40.02 (<0.001) | -38.33 (<0.001) |
|  | Lipid-lowering drugs | -45.20 (<0.001) | -54.55 (<0.001) | -48.53 (<0.001) | -50.04 (<0.001) |
|  | PPIs | 43.25 (<0.001) | -34.61 (<0.001) | -24.50 (<0.001) | -30.86 (<0.001) |
|  | Antidepressant drugs, antipsychotics, mood stabilizers drugs | -30.09 (<0.001) | -28.21 (<0.001) | -13.50 (<0.001) | -7.81 (<0.143) |
| LAGB | Glucose-lowering agents | -27.98 (<0.001) | -30.90 (<0.001) | -21.47 (<0.001) | -23.39 (<0.001) |
|  | Antihypertensive drugs | -17.73 (<0.001) | -24.75 (<0.001) | -15.59 (<0.001) | -12.38 (<0.001) |
|  | Lipid-lowering drugs | -26.56 (<0.001) | -22.54 (<0.001) | -0.64 (1.000) | 3.66 (1.000) |
|  | PPIs | 51.64 (<0.001) | -43.06 (<0.001) | -26.49 (<0.001) | -26.59 (<0.001) |
|  | Antidepressant drugs, antipsychotics, mood stabilizers drugs | -30.96 (<0.001) | -29.37 (<0.001) | -12.33 (<0.001) | -8.86 (0.032) |
| BPD | Glucose-lowering agents | -58.36 (0.001) | -66.76 (<0.001) | -66.83 (<0.001) | -75.28 (<0.001) |
|  | Antihypertensive drugs | -18.98 (0.177) | -34.05 (0.002) | -19.07 (0.181) | -32.24 (0.009) |
|  | Lipid-lowering drugs | -22.73 (1.000) | -30.50 (0.678) | -24.03 (0.999) | -24.03 (1.000) |
|  | PPIs | 62.31 (<0.001) | -10.54 (0.959) | 6.49 (1.000) | 4.95 (1.000) |
|  | Antidepressant drugs, antipsychotics, mood stabilizers drugs | -4.87 (1.000) | -15.52 (1.000) | -6.93 (1.000) | 3.97 (1.000) |

LSG: Laparoscopic Sleeve Gastrectomy; GB: Gastric Bypass; LAGB: Laparoscopic Adjustable Gastric Banding; BPD: Biliopancreatic Diversion; PPIs: Proton Pump Inhibitors

*Bonferroni correction was employed to adjust for multiple comparisons
